# Twenty-eight Consecutive Laparotomies

**Published:** 1892-03

**Authors:** Virgil O. Hardon

**Affiliations:** Professor of Obstetrics and Diseases of Women and Children, Atlanta Medical College, Atlanta, Ga.


THE ATLANTA MEDICAL AND SURGICAL JOURNAL.

VOL. IX. MARCH, 1892. No. 1.

TO CONTRIBUTORS.

Articles for publication must reach this office not later than 15th inst.; and are expected to be published exclusively in this JOURNAL. Extra copies of JOURNAL furnished contributors when requested. Reprints by special arrangement. Publication of an article does not necessarily imply endorsement of the views therein expressed.

ATLANTA MEDICAL AND SURGICAL JOURNAL, Post-office box 431.

### Original Communications.

### TWENTY-EIGHT CONSECUTIVE LAPAROTOMIES.

BY VIRGIL O. HARDON, M. D.,

Professor of Obstetrics and Diseases of Women and Children, Atlanta Medical College, Atlanta, Ga.

CASE XXIII*.—*Oöphorectomy. Recovery.* N. H., single, age 38. For about a year and a half has been conscious of a tumor in the right inguinal region. For the last five months has suffered from pelvic pain, metrorrhagia and general impairment of health. Examination showed an intramural fibroid tumor as large as an orange.

*Operation, September* 18, 1890. Present, Drs. Harris and Kennedy. Median incision, extirpation of both ovaries and tubes, which were apparently normal. Time of operation twenty-eight minutes. Uneventful recovery. Highest temperature 100.2 on

*Numbering continued from previous series.

the evening of the second day. Report from the patient two years later showed that the tumor had entirely disappeared, her health was perfectly restored and she was about to be married.

CASE XXIV.—*Myomotomy during Pregnancy. Recovery.* M. A., married, age 26. Patient when four months pregnant with her first child began to suffer with symptoms of threatened miscarriage. Examination revealed a pedunculated subserous fibroid tumor wedged between the right side of the uterus and the pelvic wall.

*Operation, September* 25, 1890. Present, Drs. Harris and Kennedy. Median incision. The tumor was dislodged from the pelvis with some difficulty, its pedicle tied in three parts and divided and the stump dropped back into the abdomen. Uneventful recovery. Highest temperature 99.6 on the evening of the second day. Pregnancy went on to full term and patient was delivered of a living child.

CASE XXV.—*Ovariotomy. Recovery.* J. H., married, age 41. Health good until two years ago, when she noticed a tumor in the left inguinal region, which increased in size up to the present time. Examination showed a cystic tumor springing from the left broad ligament, as large as a man's head.

*Operation, September* 27, 1890. Present, Drs. Harris and Kennedy. Median incision. The tumor, which was an intraligamentous monocyst, was imbedded in adhesions in its pelvic portion. The contents were evacuated and on account of adhesions it was found impossible to shell it out from the broad ligament. The abdominal portion was therefore cut away and the edges of the remaining portion stitched to the edges of the abdominal incision. The sac was packed with bichloride gauze. Slow recovery, the temperature rising to 102.4 on the third day. The cavity contracted to a small fistula which still remains. General health perfectly restored.

CASE XXVI.—*Oöphorectomy. Recovery.* S. A., married, age 32. Patient has suffered with persistent pelvic pain for several years which has failed to yield to treatment. Examination showed both ovaries to be enlarged and the left one prolapsed into Douglas' pouch.

*Operation, October* 3, 1890. Present, Drs. Harris and Kennedy. Median incision, both ovaries removed and found to be cystic. Uneventful recovery. Highest temperature 100.2. Patient restored to perfect health.

CASE XXVII.—*Oöphorectomy. Recovery.* W. C. W., married, age 23. Has suffered for two years with persistent pelvic pain, aggravated at the menstrual periods. During the last six months has been unable to walk and is bedridden. Examination showed both ovaries to be excessively tender.

*Operation, November* 4, 1890. Present, Drs. Harris and Kennedy. Median incision. Both ovaries removed and found to be cirrhotic. Uneventful recovery. Highest temperature 101.3. For a time the patient's condition was improved and she was able to walk about. After her return home, however, she took to her bed, and at last accounts was no better off than before the operation.

CASE XXVIII.—*Salpingo-oöphorectomy. Recovery.* J. O., married, age 28. Since the birth of her child, fifteen months ago, has suffered with severe pelvic pain, which has failed to yield to long continued treatment. Examination showed the ovaries and tubes to be enlarged and tender.

*Operation, November* 19, 1890. Present, Drs. Kennedy and Childs. Median incision. Both ovaries and tubes removed. The ovaries were enlarged and cystic. Salpingitis in both tubes. Adhesions were extensive and the organs were removed with difficulty. In the course of the operation the bladder was accidentally opened to the extent of an inch. The opening was closed by a Lembert suture of catgut. Rapid recovery. Highest temperature 100.8. The urine was bloody for the first twenty-four hours, and was drawn with a catheter every four hours. A drainage tube was placed in the abdomen and removed at the end of thirty hours. The patient was completely restored to health and has remained so to the present time.

CASE XXIX.—*Herniotomy. Recovery.* L. B., married, age 39. This is the same patient as Case XIII., previously reported as having been left with a ventral hernia after an oöphorectomy for prolapsed ovary. The hernia increased in size until it rendered her unable to work.

*Operation, November* 25, 1890. Present, Dr. Kennedy and the students of the Atlanta Medical College. Incision an inch to the left of former one over the most prominent part of the hernia. The recti muscles were found to be separated for a distance of two inches, leaving only a covering of skin, peritoneum and cicatricial tissue over the intestines. These were divided. Silk sutures were then passed through the whole thickness of the abdominal wall. Before tying them the muscles were drawn together and united by a continuous suture of strong catgut. The silk sutures were then tied. Recovery rapid. Highest temperature 100.4. Firm adhesion of the muscles took place and there has been no return of the hernia.

CASE XXX.—*Oöphorectomy. Recovery.* W. M., married, age 18. Since a severe fall two years ago patient has suffered severe pain in the left inguinal region, which has completely disabled her and which has failed to yield to treatment. Examination showed the left ovary to be much enlarged and excessively tender.

*Operation, November* 29, 1890. Present, Drs. Harris and Kennedy. Median incision. The left ovary was removed and found to be enlarged, completely disorganized and reduced to a soft pulp. As the right ovary was perfectly normal it was allowed to remain in accordance with the request of the patient. Uneventful recovery. Highest temperature 101.6. Patient has remained in good health up to the present time.

CASE XXXI.—*Exploratory Incision. Recovery.* J. P., single, age 24. About six months ago begun to notice a symmetrical enlargement of the abdomen which has steadily increased until it has reached the size of about the seventh month of pregnancy. The most careful examination of the heart, liver and kidneys revealed no cause of the enlargement. There was no œdema of any part of the body. The general health was somewhat impaired. The abdomen was found, upon examination, to be filled with ascitic fluid. On either side of the womb were felt two irregular, indistinct masses.

*Operation, December* 8, 1890. Present, Drs. Todd, Harris and Kennedy. Median incision allowed the escape of a large quantity of ascitic fluid tinged with blood. On each side of the womb were found large cancerous masses which involved the ovaries, tubes, broad ligaments and fundus uteri. The parietal and visceral peritoneum were also studded with cancerous deposit. The abdominal incision was closed without further operation. Patient made a good recovery from the operation. In about three weeks the fluid had reaccumulated and was drawn off through a trocar. The patient returned to her home in Alabama, where she died the following April.

CASE XXXII.—*Oöphorectomy. Death.* A. B., married, age 40. Has suffered for over three years with persistent pelvic pain which completely disabled her. Has acquired the morphine habit and takes from ten to fifteen grains of the drug every day. Examination showed the ovaries to be tender and enlarged. Patient in bad general health.

*Operation, December* 10, 1890. Present, Drs. Kennedy, Richardson and the students of the Atlanta Medical College. Median incision. No adhesions. Ovaries enlarged and cystic. Operation completed in nineteen minutes. Patient did badly from the start. Her temperature did not rise above 101, but her strength gradually failed and she died of exhaustion on the seventh day.

CASE XXXIII.—*Salpingo-oöphorectomy. Recovery.* W. M., married, age 34, had suffered for several years from recurrent attacks of pelvic peritonitis, which had seriously impaired her health. Examination showed a tumor as large as a hen's egg on the left side of the womb.

*Operation, December* 12, 1890. Present, Drs. Kennedy, Childs and Greenwood. Median incision. The tumor was found to be the left tube filled with pus. It was imbedded in adhesions and with its corresponding ovary was removed with difficulty. The right ovary and tube were also removed. Drainage for twenty-four hours. Good recovery. Highest temperature 100.6. Has remained in good health up to the present time.

CASE XXXIV.—*Secondary Laparotomy. Recovery.* E. R., single, age 20. Patient was operated on eighteen months ago by another operator and both ovaries removed for persistent pelvic pain. The pain was relieved for about six months and then returned as severely as before. Examination revealed nothing to account for the pain.

*Operation, January* 31, 1891. Present, Drs. Kennedy and Childs. Median incision. The stumps of both ovaries were found adherent to the small intestines. No other abnormal condition. The adhesions were divided with scissors. Good recovery. Highest temperature 99.8. The pain was relieved for several months and the patient was apparently restored to health. She is now, however, under the treatment of another physician for pelvic abscess.

CASE XXXV.—*Hysterorraphy. Recovery.* C. M., married, age 38. Has suffered from complete prolapse of the womb since the birth of her last child, two years ago, and is disabled from work by it.

*Operation, February* 3, 1891. Present, Drs. Kennedy, Childs, Burton of Valdosta, and Smith of Thomasville. Median incision. The uterus was brought up to the incision and stitched to the abdominal wall by catgut ligatures passed through the horns of the uterus and the walls of the abdomen. Good recovery. Highest temperature 101. The womb has remained in place up to the present time. She has since developed a cancer of the cervix which will ultimately prove fatal.

CASE XXXVI.—*Salpingo-oöphorectomy. Recovery.* A. L., married, age 35. Severe pain with recurrent attacks of pelvic peritonitis. Examination showed both tubes distended by fluid and immovably imbedded in adhesions.

*Operation, February* 4, 1891. Present, Drs. Kennedy and Childs. Median incision. Both tubes were imbedded in plastic exudation and both were distended, the right with pus to the size of a hen's egg, the left with blood to the size of an English walnut. Both were removed with their accompanying ovaries. Drainage for thirty-six hours. Slow recovery. In the track of the drainage tube a fistula remained which diminished to the depth of two and a half inches and the size of a small probe. Patient went home in this condition and has not since been heard from.

CASE XXXVII.—*Oöphorectomy. Recovery.* B. J., married, age 36. For about three years patient has felt a hard tumor in the lower part of the abdomen which has given her no trouble until about a year ago, since which time she has suffered from profuse metrorrhagia and severe pain. Examination shows an intramural fibroid tumor as large as an infant's head.

*Operation, February* 21, 1891. Present, Drs. Kendrick, Kennedy and Childs. Median incision. Both ovaries removed and found to be normal. Rapid recovery. Highest temperature 99.7. The pain and hemorrhage have not returned up to the present time, and the tumor is notably diminished in size.

CASE XXXVIII.—*Ovariotomy. Recovery.* W. E., married, age 36. Has suffered for a year and a half from pelvic pain and a feeling of pressure in the pelvis. Examination revealed a cystic tumor as large as a Tangerine orange on the left size of the pelvis.

*Operation, March* 3, 1891. Present, Drs. Kennedy and Childs. Median incision. The tumor was found to be a cyst of the left ovary and was removed. The right ovary was also removed. Uneventful recovery. Highest temperature 100.4. Patient has remained in perfect health up to the present time.

CASE XXXIX.—*Ovariotomy. Recovery.* M. W., single, age 25. For about a year has suffered from pelvic pain and a sense of pressure in the pelvis. Examination revealed a cystic tumor on the left side as large as an orange.

*Operation, March* 6, 1891. Present, Drs. Kennedy and Childs. Median incision. The tumor was found to be a cyst of the left ovary and was easily removed as there were no adhesions. Rapid recovery. Highest temperature 99.8. Patient returned to her home in Florida on the twentieth day.

CASE XL.—*Laparotomy for Intestinal Obstruction. Death.* C. B., married, age 42. In good health until a week ago, but habitually constipated. After an attack of constipation the bowels began to swell and become painful with high fever. Vomiting followed and became stercoraceous. When seen by me was almost in collapse.

*Operation, March* 11, 1891. Present, Drs. Childs, Kennedy, Ebbert and Smith. Median incision. Examination of the intestines revealed after careful search an acute flexure of the ascending colon just above the ileo-cæcal valve, caused by a band of lymph. The band was divided and the bowel straightened out and returned to the abdomen. There was a high degree of inflammation at the point of obstruction but no strangulation. After the operation the condition of the patient did not improve, the bowels still failed to act and she died on the third day.

CASE XLI.—*Salpingo-oöphorectomy. Recovery.* L. J., married, age 36. Persistent pelvic pain for two years, aggravated at the menstrual periods. General condition good but is disabled from work by the pain. Examination showed both ovaries to be enlarged and the right one prolapsed.

*Operation, April* 27, 1891. Present, Drs. Kennedy, Childs and McRae. Median incision. Both ovaries and tubes were removed. The ovaries were enlarged and cystic and the tubes thickened. Uneventful recovery. Highest temperature 99.8. Patient returned to her home entirely relieved; and six months later reported that she still remained well and was able to work.

CASE XLII.—*Oöphorectomy. Death.* W. P., married, age 26. Suffered for several years with persistent pelvic pain aggravated by exercise. No relief from treatment. Examination showed the left ovary prolapsed into Douglas' pouch, and excessively tender.

*Operation, May* 11, 1891. Present, Drs. Kennedy and Childs. Median incision. Both ovaries removed. Patient did well for two days, when her temperature rose to 102, the abdomen began to swell and vomiting ensued. Vigorous efforts at catharsis failed to produce action of the bowels, and she died on the fifth day apparently from intestinal obstruction.

CASE XLIII.—*Salpingo oöphorectomy. Death.* J. M., married age 24. Persistent pelvic pain with recurrent attacks of pelvic peritonitis. Examination showed both tubes to be distended with fluid.

*Operation, June* 4, 1891. Present, Drs. Kennedy and Childs. Median incision. Both tubes were found to contain pus and were imbedded in adhesions. They were removed with difficulty, together with the ovaries. Considerable hemorrhage, which was controlled by hot water and sponge packing. A glass drainage tube was left in the wound. The patient was very willful and uncontrollable. She did well until the evening of the second day, when, in the momentary absence of her nurse, she turned violently over on her face. Soon after she complained of faintness and blood was found to be escaping in large quantities through the drainage tube. I was sent for at once, but when I arrived she was pulseless and died in a short time with the symptoms of acute hemorrhage. The drainage tube was withdrawn and found intact, but its pressure, when she turned on her face, had undoubtedly ruptured some large blood-vessel.

CASE XLIV.—*Exploratory Incision. Recovery.* A. B., married, age 26. For eight months has suffered from profuse metrorrhagia which has reduced her strength. Health good in other respects. No pelvic pain. Examination revealed an intramural fibroid tumor as large as a goose egg.

*Operation, June* 9, 1891. Present, Drs. Kennedy and Childs· Median incision. The ovaries, tubes and uterus were found in extricably matted together in a mass of plastic exudation, so that the removal of the ovaries was deemed impossible. The incision was closed and the patient made a rapid recovery with scarcely any rise of temperature. Strange to say, the metrorrhagia did not return after the operation and has not recurred up to the present time.

CASE XLV.—*Oöphorectomy. Recovery.* F. P., married, age 32. Patient has suffered since her marriage two years ago with persistent pelvic pain, with marked nervous symptoms amounting almost to insanity. Examination showed a prolapsed ovary, enlarged and tender.

*Operation, August* 20, 1891. Present, Drs. Kennedy and Childs. Median incision. Both ovaries removed. Rapid recovery. Highest temperature 100.2. The pain and nervous symptoms have entirely ceased, and the patient has remained in perfect health up to the present time.

CASE XLVI.—*Oöphorectomy. Recovery.* H. J., married, age 29. Persistent pelvic pain which failed to yield to treatment. Examination showed both ovaries enlarged.

*Operation, September* 19, 1891. Present, Drs. Kennedy and McRae. Median incision. Both ovaries removed and found to be cystic. Uneventful recovery. Pelvic pain entirely and permanently relieved. Owing to neglect to wear an abdominal bandage after getting up the patient has a small ventral hernia.

Case XLVII.—*Oöphorectomy. Recovery.* M. P., single, age 35. For several years has had epileptic convulsions, occurring only at the menstrual periods. She is physically a wreck, with mind seriously impaired. Examination revealed nothing abnormal in the pelvis.

*Operation, October* 8, 1891. Present, Drs. Kennedy and Childs. Median incision. Both ovaries removed, apparently normal. Recovery uneventful except a small stitch abscess. Highest temperature 101.2. Since the operation there has been no recurrence of the epileptic seizures. Her general health has improved, but her mental condition remains unchanged.

Case XLVIII.—*Oöphorectomy. Death.* E. B., single, age 38. Has suffered for several years with severe pelvic pain, which of late has become unbearable, so that she has resorted to anodynes and has acquired the morphine habit. Examination showed both ovaries to be enlarged and tender. General condition decidedly bad.

*Operation October* 10, 1891. Present, Drs. Kennedy and Childs. Median incision. Both ovaries removed. Patient did badly from the start. Abdominal distention and vomiting of bile occurred on the second day. All attempts at catharsis were ineffectual, and she died of peritonitis on the morning of the fourth day.

Case XLIX.—*Laparotomy for Pelvic Abscess. Recovery.* Patient began to suffer pelvic pain about eight months ago. Three months ago she began to have rigors and fever and noticed a lump above the pubes a little to the right of the median line. Examination showed a tumor as large as an infant's head, apparently connected with the womb and feeling like a fibroid tumor. No fluctuation was detected.

*Operation, January* 27, 1892. Present, Drs. Childs and Johnson. Median incision. Upon opening the abdomen the tumor was found to contain fluid and to be universally adherent to surrounding organs and to the abdominal wall. An incision into the tumor allowed the escape of about a quart of pus. The sac was washed out with an antiseptic solution and a rubber drainage tube inserted, which was removed on the third day. It was afterwards washed out daily and packed with corrosive sublimate gauze. By contraction and granulation it is now reduced to a small sinus about a half an inch deep, which is rapidly healing. The patient has gained flesh and strength and now feels perfectly well.

CASE L.—*Oöphörectomy. Recovery.* C. B., married, age 32. Has suffered for two years with constant pelvic pain which has completely disabled her. She has been under treatment during the most of that time without any relief. Examination shows prolapse of both ovaries.

*Operation, January* 30, 1892. Present, Drs. Childs and Johnson. Median incision. Both ovaries removed. Uneventful recovery. Highest temperature 100. Patient returned home on the twentieth day entirely free from pain.

